# Supplementary material for: Are carbon emissions trading and green financial instruments synergistic? -Comprehensive quantitative research based on content analysis
Source: PLoS One. 2024 Mar 7;19(3):e0298601. doi: 10.1371/journal.pone.0298601 (PMC10919601; doi:10.1371/journal.pone.0298601)
Supplement: S1 Data — (ZIP) [file pone.0298601.s001.zip › Data/地级市Z_F工作报告.zip!/╣π╢1⁄2/╤⌠╜¡╩╨/└┤╫╘╣1⁄2╓┌║┼í╛┬φ┐╦╩2╛▌═°í┐_2018.docx]

各位代表：

　　现在，我代表市人民政府，向大会报告政府工作，请予审议，并请政协委员和列席人员提出意见。

　　一、2017年工作回顾

　　刚刚过去的一年，是本届政府谋篇布局、开拓创新、真抓实干、砥砺奋进的一年。在省委、省政府和市委的正确领导下，市政府深入贯彻落实党的十八大、十八届历次全会精神，认真学习贯彻习近平新时代中国特色社会主义思想和党的十九大精神，按照习近平总书记对广东工作重要指示批示要求，全面贯彻落实省第十二次党代会和省委十二届二次全会精神，紧紧围绕市第七次党代会提出“以海兴市、绿色发展，决胜全面小康、建设富美阳江”的战略部署，实施“六大举措”，建设“六个新城”，基本完成了市七届人大一次会议确定的各项目标任务。预计全市地区生产总值1428亿元，增长6.8%;地方一般公共预算收入60.66亿元，增长7.1%;规模以上工业增加值507亿元，增长9%;固定资产投资544亿元，增长8%;社会消费品零售总额691亿元，增长8.9%;城镇居民人均可支配收入27400元，增长8.5%;农村居民人均可支配收入15500元，增长11.3%。

　　(一)经济发展稳中向好。先后召开“以海兴市”大会、招商引资大会、“三大抓手”推进大会和旅游发展大会，统一思想、凝聚共识，明确目标、奋力推进，举全市之力打赢“三大硬仗”和“三个大仗”，全市经济运行触底回升、逐季向好，发展质量逐步提高、持续优化。地区生产总值在上半年扭转了自2015年底开始连续15个月下滑的局面，固定资产投资、外贸进出口增长扭负为正，财政收入结构得到优化，招商引资势头日趋良好。新增阳春新钢铁、阳江核电两家年产值超100亿元企业，广青金属科技上榜广东企业500强，并与阳春新钢铁进入广东制造业100强。首批24个总投资279亿元的PPP项目全面启动，阳春凌霄泵业成为粤西首家上市装备制造企业，英格电气、阳江港港务在新三板挂牌上市。率先在粤东西北地区试点推行“证照分离”改革，新登记企业增长11.5%。

　　(二)产业结构优化升级。三大主导产业取得突破性进展，产业新城建设成效显著。不锈钢全产业链加快完善，投资20亿元的广青热轧项目开工建设，投资22亿元的甬金冷轧项目成功签约，粤泰金属制管等一批不锈钢下游项目正在洽谈或落户。风电产业集群加速形成，明阳主机等4个总投资31亿元的风电装备制造项目落户建设，计划投资29亿元的中车电机等14个配套项目陆续进驻;中广核南鹏岛等首批4个总投资260亿元、装机容量130万千瓦的海上风电项目全部核准动工。食品加工业加快发展，成功引进投资7亿元的亨氏食品、投资30亿元的和鲜食品等项目，投资16亿元的美味鲜等项目建设扎实推进。园区经济不断壮大，园区规上工业产值占全市规上工业总产值54.2%。成立市招商局和高端不锈钢、先进装备制造两支招商小分队，引进国信通等项目104个、总投资461.9亿元。全面启动创建国家高新区，新认定国家高新技术企业14家，新增省级工程研究中心4家，年产值5亿元以上企业实现研发机构全覆盖。签约优质旅游项目16个、总投资506亿元，阳东新洲地热小镇上榜广东特色小镇创建示范点。农业供给侧结构性改革深入推进，新增省级名牌农产品9个，青洲岛等4处海域列入国家级海洋牧场示范区建设。

　　(三)基础设施建设提速。珠海(阳江)合作共建园建设全面铺开、快速推进，创新交流中心等一批项目建成，疏港大道南等一批项目加快建设，生活配套区首期等一批项目加快前期工作，园区面貌发生显著变化。推进以“一港一路一桥”为重点的交通基础设施建设，阳江港总体规划审批实现突破，4#、13#-14#等码头泊位完成主体工程或试运营，阳江港至珠海高栏港集装箱班轮航线正式开通，汕湛高速阳春松柏至八甲段建成通车，阳江合山机场成为全省第3家可对公众开放的通用机场，深茂铁路阳江段完成铺轨工程，海陵岛大桥、中阳高速阳春段加快建设，G325北惯至白沙段改线、沈海高速阳江段四改八扩建工程正式动工，广东滨海旅游公路阳江雅韶至溪头段(含阳江港大桥)、广湛客运专线前期工作扎实推进。投入13.1亿元完成一批电网基建工程，220千伏旗鼓岭等15个重要输变电工程竣工运营。双捷拦河闸重建工程投入运行，海堤达标加固123.7公里，大河水库引水工程前期工作积极推进。着眼打基础、利长远，谋划了241个总投资974亿元的“三大抓手”基础设施项目。

　　(四)城乡面貌显著提升。加快推进城市向南向海发展，滨海新城和滨海生态公园启动规划建设，连围河整治工程全面推进，金平路一期、深茂铁路阳江站站前路等一批市政道路加快建设，阳江职业技术学院新校区选址确定并启动前期工作，阳江国际金融中心即将建成，市妇幼保健院新院投入使用;市会展中心等一批城市配套设施建成启用，始兴路改造等一批市政道路竣工通车，森林公园一期、漠阳湖公园一期、南山公园等一批城市公园基本建成，北湖公园改造正式启动。

　　成功创建国家卫生城市，创美了环境，创出了精气神，人民群众对阳江更有认同感、归属感和自豪感。建立健全城市管理考核机制，坚持每月一考核两月一点评，城市管理逐步实现网格化精细化。全面启动创建国家森林城市和全国文明城市，两个镇入选广东省森林小镇，两条村获评“全国文明村”荣誉称号。农村生活垃圾保洁机制基本建立，88条省定贫困村1269条20户以上自然村全部完成“三清理三拆除三整治”，3个省级新农村示范片进展顺利，100条美丽乡村如期建成。深入创建国家环保模范城市，全面建立市县镇村四级河长制、湖长制，加强大气、水、土壤污染防治，城市空气质量保持优良，漠阳江水环境质量总体为优，饮用水源水质达标率100%，土壤总体质量状况良好，生态新城建设取得良好成效。

　　(五)人民生活持续改善。制定实施全面建成小康社会补短板行动方案。省市十件民生实事全面完成，民生支出占地方一般公共预算支出的比重达67.5%。城镇新增就业3.93万人，城镇登记失业率控制在3%以内，城镇职工五大险种新增参保10.18万人次。底线民生保障水平巩固提升，残疾人“两项补贴”持续提高，全市失能特困老人集中治疗和护理工作扎实推进。教育现代化先进市建设加快，幼儿园办园水平和保教质量不断提升，改善贫困地区义务教育薄弱学校基本办学条件项目全面实施，市一级标准以上公办普通高中学校占比达100%，中职毕业生就业率达97%以上，困难家庭学生资助实现从学前教育到高等教育全覆盖。两家镇中心卫生院启动升级建设，8家镇卫生院和99个村卫生站完成标准化建设。取消药品加成，与全省404家医院实现省内异地就医直接结算、费用统一清算。新建31个省级、20个市级基层文化服务中心，完成“南海I号”申遗保护展示工程，成功举办风筝文化节、海陵岛环岛国际马拉松赛和首届“蚝美阳江”美食节等活动。深入推进脱贫攻坚，2.864万相对贫困人口实现脱贫。建成公租房356套，完成农村危房改造6300户。新增100辆新能源公交车。加强综治信访维稳工作，有效遏制各类违法犯罪，强化安全生产和食品药品监管，人民群众的获得感、幸福感、安全感不断增强。

　　(六)政府治理规范有效。加强法治政府建设，2016年度依法行政考评在粤东西北地区排名第一、全省第八。深化“放管服”改革，出台公布市政府部门行政审批中介服务目录及事项清单，取消市县两级61项行政许可事项。推进“一门式一网式”政府服务模式改革及行政审批标准化，网上办事大厅联通52个市级部门、6个县(市、区)政府(管委会)及其部门，开通48个镇街网上办事站、826个村居网上办事点，群众和企业办事更加方便。优化行政审批流程，工业及房地产项目审批时限由156个工作日压减到86个工作日。认真执行市人大及其常委会决定，坚持向市政协通报情况，自觉接受监督。办理人大代表议案和建议62件，政协建议案和提案188件，办复率均为100%。坚决执行中央八项规定及其实施细则，认真落实党风廉政建设责任制，加强政纪监督、法律监督和审计监督，查处了一批违纪违法案件，团结务实、廉洁高效的干事氛围更加浓厚。

　　与此同时，双拥共建、民族宗教、外事侨务、对台、人防、地震、供销、档案、地方志、妇女儿童等工作取得新成绩。

　　各位代表!过去一年取得的成绩，是市委统揽全局、正确领导的结果，是市人大、市政协大力支持和有效监督的结果，是全市人民共同努力的结果。在此，我代表市人民政府，向全市广大干部群众，向各民主党派、工商联、无党派人士，向驻阳江部队、武警官兵、中央和省驻阳江单位，向离退休老同志，向关心支持阳江改革发展的社会各界人士，表示衷心感谢!

　　在肯定成绩的同时，我们也清醒地看到，发展中还存在不少困难和问题：一是经济总量偏小。我市地区生产总值虽超千亿元，但经济总量小、底子薄、实力弱的状况没有根本改变，做大规模总量和提升发展质量的双重任务仍然艰巨繁重。二是经济增长乏力。重大项目特别是大型产业项目较少，在建重大能源项目投资逐年下降，新引进的工业大项目和实施的基础设施项目大多处于前期或初建阶段，新的经济增长点还不多，经济持续稳定向好的基础还不牢固。三是现代产业比重较低。一产偏高、二产不强、三产滞后问题依然突出，新兴产业发展较慢，高新技术产业占比较小，现代服务业比重不高，产业总体处于中低端。四是创新能力弱。企业整体自主创新能力不强，科技企业孵化器建设滞后，国家级研发平台基本空白。五是改善民生任务艰巨。虽然农村贫困人口累计脱贫率等10项民生指标基本达到或接近目标，但教育、医疗、文化等基本公共服务与群众期望还有较大差距。六是政府效能有待提升。行政审批效率仍然不高，基层治理能力较为薄弱，一些干部业务能力、责任意识、担当意识不强，不敢为、不善为、慢作为的现象仍然存在，党风廉政建设任务依然艰巨。对这些问题，我们一定高度重视、认真研究，努力把政府工作做得更好，绝不辜负人民群众的期望!

　　二、2018年工作安排

　　今年是阳江建市30周年，是全面贯彻落实党的十九大精神的开局之年，是改革开放40周年，是决胜全面建成小康社会、实施“十三五”规划承上启下的关键一年。做好今年的工作，意义十分重大。政府工作的总体要求是：深入学习贯彻习近平新时代中国特色社会主义思想和党的十九大精神，坚持以习近平总书记对广东工作重要指示批示精神为统领，坚持稳中求进工作总基调，坚持新发展理念，按照高质量发展的要求，统筹推进“五位一体”总体布局和协调推进“四个全面”战略布局，紧扣中央经济工作会议、中央农村工作会议和省委十二届二次、三次全会及市委七届四次、五次全会部署，深入推进供给侧结构性改革，突出以海兴市、绿色发展，打好“三大攻坚战”，实施“六大举措”，建设“六个新城”，以项目落实为重要抓手，促进经济健康发展、社会和谐稳定，加快全面建成小康社会。

　　根据中央和省的政策导向，着眼于高质量发展和加快发展的现实需要，着眼于经济发展和民生改善的同步推进，市委、市政府研究提出，今年经济社会发展主要预期目标是：地区生产总值增长6.5%左右;地方一般公共预算收入增长7%;规模以上工业增加值增长7.5%左右;固定资产投资增长8%;进出口总额增长2%;社会消费品零售总额增长8.5%;居民消费价格涨幅控制在3%以内;居民人均可支配收入增长高于经济增长，城镇登记失业率控制在3%以内，完成节能减排约束性指标任务。

　　为实现上述目标，我们将重点抓好八个方面工作：

　　(一)促进经济稳定增长

　　加大招商引资力度。坚持把招商引资作为经济工作的“一号工程”来抓，力争全年引进项目100个以上、总投资450亿元以上，其中超10亿元项目10个以上。发挥资源、产业优势，强化精准招商和招商选资，重点面向广州、深圳、佛山等珠三角城市开展招商，着力引进规模大、效益好、带动力强的项目。压实各县(市、区)和园区招商引资主体责任，发挥市招商局和两支招商小分队主力军作用，重点依托广青金属科技、三峡集团、明阳集团、厨邦食品等企业开展高端不锈钢、风电装备制造、食品加工等产业链招商，引进关键环节、核心企业和上下游配套企业，实现引进一个、带来一批，努力形成产业集群。推行项目负责人制，加强招商项目落地全过程无缝对接服务，提高合同履约率、资金到位率、项目开工率。召开“以海兴市”工作现场会，出台优化营商环境扶持企业发展的政策措施，注重招商与亲商、扶商、安商有机结合，建立亲清新型政商关系。

　　持续扩大有效投资。围绕重大产业项目和“三大抓手”基础设施，突出重点，强化措施，全力推进年度投资243亿元的103个市重点项目建设，力促年度投资51.5亿元的21个项目建成投产，推动年度投资149.8亿元的48个续建项目加快建设，确保年度投资41.7亿元的34个项目全面动工;深入开展“三大抓手”基础设施大会战，重点突破53个交通项目、133个市政项目和49个园区基础设施项目，确保完成“三大抓手”基础设施投资181.5亿元以上;全面动工首批总投资279亿元的24个PPP项目，加快第二批总投资143.2亿元的12个PPP项目前期工作。落实项目清单管理制度，压实市直部门和县(市、区)政府责任。实行重点项目征地拆迁市领导挂点制度，着力解决项目推进瓶颈问题。加强项目督查，定期通报项目进展情况，鞭策后进、激励先进，确保完成全年投资目标任务。

　　努力挖掘消费潜力。促进居民增收，加强市场监管，营造良好的消费环境。加快服务业模式创新和跨界融合，推动东汇城、盈信广场、星港汇等城市综合体建成开业，培育15家限上商贸企业。发挥市会展中心平台作用，大力发展家居建材博览会等会展经济，促进居民消费。实施“互联网+”流通行动计划，培育发展本土电商标杆企业和园区，新建200家农村电商服务站点。加快国际汽车商贸城、城北物流园等项目建设，努力把城北打造成汽车贸易、现代物流集聚区。大力发展海路货客运，提高货物周转量。

　　加快外贸转型发展。着力扩大出口，办好第17届刀博会，组织企业参加广交会等30场境内外展会，促企业抢订单、拓市场。大力培育外贸新业态，推动阳江(国际)五金刀剪商贸城纳入国家市场采购贸易方式试点，鼓励外贸企业构建海外自主营销体系。积极扩大进口，引导企业在阳江口岸加大原材料、先进设备、关键零部件等产品进口。加快口岸通关改革创新，推行大海关监管模式，推进我市国际贸易“单一窗口”国家标准版扩大试点，提升外贸便利化程度。推动阳江港公司化运营管理，完善港口配套设施，尽快开通集装箱外贸航线货轮运输定期航班，降低企业经营成本。

　　提高金融服务水平。加强金融政策与产业政策对接，提高金融服务实体经济水平。稳步推进金融监管体制改革，加强小额贷款公司等7类金融机构监管，严厉打击非法集资行为，推动经济去杠杆，确保辖区内地方法人银行机构杠杆率达到4%的监管最低要求。全面完成农商行组建工作，争取一批金融机构在我市新设立分支机构，优化信贷结构和金融产品。鼓励支持企业到“新三板”“新四板”挂牌，到创业板和中小板、主板上市。推进广东省科技金融综合服务中心阳江分中心建设，加强融资贷款、产权交易、股权投资等服务。

　　(二)推进产业转型升级

　　深化供给侧结构性改革。按照“破”“立”“降”的要求，深入推进“三去一降一补”，推动经济发展质量变革、效率变革、动力变革，提高全要素生产率。落实省、市“实体经济十条”，大力降低实体经济成本，全面提升实体经济规模与效益。加快建设具有阳江特色的现代化经济体系，落实《广东省沿海经济带综合发展规划(2017-2030)》和《珠江西岸先进装备制造产业带聚焦攻坚计划(2018-2020)》，发挥阳江作为珠三角辐射粤西的战略支点优势，争取谋划布局省级产业集中集聚区，建设珠江西岸产业转移主承接区，着力打造高端不锈钢、电力能源、装备制造、食品加工等一批年产值超500亿元产业集群。实施大型骨干企业培育工程，推动广青金属科技年产值达200亿元，新增年产值50亿元以上企业1家、10亿元以上企业3家。推动五金刀剪等传统产业优化升级，集中资源扶持2-3家五金刀剪企业，在材料、工艺、设计、品牌等方面攻坚发力，提升阳江五金刀剪企业在国内外市场的话语权。

　　做大做强工业经济。依托阳江高新区等重大发展平台，着力建设沿海临港工业重要基地。全力以赴加快高端不锈钢、海上风电两大主导产业核心项目及关联配套项目建设。加快广青热轧项目建设进度，力促甬金冷轧项目上半年动工，推进不锈钢热轧、冷轧下游产业发展，完善不锈钢全产业链。推动4个风电装备制造项目建成投产，其中明阳风机叶片项目7月底投产、明阳主机项目9月底投产;促进首批4个海上风电项目在年中全面开展海上风机吊装施工，力争第二批170万千瓦海上风电项目年内全部核准。争取在2月设立首期规模60亿元的海上风电产业发展基金，扶持海上风电产业尽快做大规模。围绕新能源基地建设，推动阳江核电站5号机组7月投产，促进阳江抽水蓄能电站、LNG调峰储气库及配套等项目加快建设，谋划发展“储能”等新型能源产业。力促亨氏食品、和鲜食品等项目加快动工，推进美味鲜等项目建设，壮大食品加工业。力争维达纸业项目5月底前投产，扎实推进华正能电缆、国信通、新兴铸管二期等一批项目，增强临港工业支撑。加快园区扩能增效，推动不少于20个投资超亿元工业项目在共建园区动工建设。

　　积极发展全域旅游。坚持全市一盘棋，以海陵岛创建国家全域旅游示范区为带动，加快建设国内外知名休闲度假胜地。编制全域旅游发展规划，实施旅游“一轴三带”空间布局。推动海陵岛十里银滩创建国家级旅游度假区，支持海陵岛红树林湿地公园、阳东北桂生态园等创建3A以上景区，建设阳春马兰风光等一批乡村旅游示范点。跟踪落实去年旅游发展大会签约的16个重点旅游项目，力争滨海新城特色主题小镇上半年动工。落实与东北和西南地区城市签订的合作框架协议，实施“互换冬天”计划。

　　(三)提升城市发展水平

　　构筑立体交通网络。积极推进融入珠三角、联接大西南的交通网络建设，提升区位优势。高速公路方面，建成汕湛高速阳春段，全面开工建设沈海高速阳江段四改八工程，加快建设怀阳高速海陵岛大桥、中阳高速阳春段，谋划推动中茂高速(粤桂界)阳春段、西部沿海高速阳江南联络线前期工作。干线公路方面，动工建设广东滨海旅游公路雅韶至溪头段(含阳江港大桥)，加快建设G325北惯至白沙段改线、G325阳西县城过境段改线和S277海陵大堤至闸坡段等项目。铁路方面，确保深茂铁路阳江段6月底前建成通车，积极谋划广湛客运专线、海陵岛轻轨、春罗铁路改造、三茂铁路阳春段改造等项目。港口方面，科学规划码头功能布局，加快阳江港进港航道改造工程及13#-14#、18#、22#等一批码头泊位建设，谋划建设丰头片区深水码头泊位，开展阳江港综合保税区、B型物流中心可研等前期工作。机场方面，积极推进阳江民用运输机场规划建设。

　　推进城区扩容提质。加快推进“多规合一”，完善各项规划编制，实现中心城区控规全覆盖。全面推动中心城区“三横四纵”城市快速路网建设，建成深茂铁路阳江站综合交通枢纽工程、“两江四岸”北岸生态湿地公园和金平路一期、城南片区“六路”等市政道路，推进城南商务集聚区综合开发项目建设，启动滨海生态公园、森林公园二期、文化公园、儿童公园、共青湖公园和第二批10个社区休闲体育公园建设。统筹阳东区和江城区的基础设施，加快贯通江城区与阳东区、建成区与新城区道路网络，全面启动S540线雅韶至白沙段扩建、阳东江台路南延线、西平路北段、三环南路改造等道路项目建设，进一步完善城市设施配套。扎实推进地下管线普查和城市地下综合管廊建设，实施城南片区“海绵城市”试点项目。积极破解旧城区停车难问题，探索建设开放街区，新建改建一批城市公厕，加强城中村及城乡结合部道路硬底化建设，改善旧城区人居环境。加快阳春市区、阳西县城、海陵区三大城市副中心和中心镇建设，完善市政基础设施和公共服务设施配套，打造一批功能完善、特色明显、环境优美、生态宜居的特色城镇。通过持续努力，把阳江建设得更宜居、更富魅力，让街区可休闲，海边可漫步，城市始终有温度。

　　加强城市精细管理。市区要进一步巩固国家卫生城市创建成果，深入推进城市综合管理执法体制改革，推动城市管理领域相关行政处罚权的集中行使，加快城市管理走向城市治理;建立完善区、街道、社区各层级网格单元，全面落实网格化管理;推进数字城管平台建设，实现市、区联网对接，提高城市智慧化管理水平;坚决治理城市管理顽疾，强力推进控违拆违，有效治理交通拥堵等城市病，使城市既有“面子”更有“里子”。阳春要全面推进国家卫生城市创建工作;阳西要启动创建省卫生县城;海陵要按更高标准抓好卫生环境整治，并结合创建活动、整治行动，提高精细化管理水平。

　　(四)实施乡村振兴战略

　　促进农业提质增效。大力推进农业供给侧结构性改革，抓好农村集体产权制度改革试点，完成农村土地确权工作，推进农业标准化生产和品牌创建，增加“三品一标”优质农产品供给，加快构建现代农业产业体系、生产体系、经营体系。落实粮食安全政府责任制，稳定全年粮食播种面积220万亩以上。抓好4个省级现代农业“五位一体”示范基地项目和“菜篮子”工程建设，保持特色优势农作物种植面积300万亩以上。鼓励发展适度规模经营，新增市级以上农业龙头企业5家。培育发展休闲农业，促进农业、林业与旅游、文化、康养融合。大力发展现代渔业，建造7艘南沙骨干船和89艘钢质渔船，建成两个休闲渔业示范基地，完成闸坡、溪头渔港项目建设，推动东平国家中心渔港项目二期、对岸渔港及配套设施项目动工。发展农海产品深加工，提高产品附加值。

　　抓好新农村建设。全域开展农村生态环境综合整治，年底前完成全市村庄人居环境基础整治任务80%以上。重点抓好农村危破旧房清拆、垃圾污水处理、“四好农村路”建设、“厕所革命”等工作，完成3946户农村危房改造，实现全市710条行政村全部通客运班线，解决涉及32万人的村村通自来水问题，农村生活垃圾有效处理率达90%。推进88条省定贫困村创建新农村示范村，扎实做好村道巷道硬底化等基础性工作，逐步全域推进创建新农村示范村。继续提升阳东、阳西省级新农村示范片，加快推进海陵、阳春示范片建设。开展移风易俗行动，不断提高乡村社会文明程度，使50%以上行政村基本达到文明村创建标准，80%以上行政村基本达到民主法治村创建标准。

　　决战精准脱贫攻坚。提高政治站位，把脱贫攻坚作为重大政治任务，强化脱贫攻坚责任和监督，开展扶贫领域腐败和作风问题专项治理，严格执纪问责，坚决打好精准脱贫攻坚战，确保小康路上一个都不掉队。抓好扶贫惠农政策落实，投入3亿元专项资金，完成2.5万相对贫困人口脱贫任务，稳定实现“两不愁、三保障”。着力推进产业扶贫、就业扶贫、金融扶贫，加大劳动力技能培训、转移就业工作力度，促进贫困户增收脱贫。坚持精准扶贫、精准脱贫，注重扶贫与扶志、扶智相结合，巩固扶贫成果，提高扶贫质量。

　　(五)推动创新驱动发展

　　创建国家高新区。围绕国家高新区产业、科技、新城三大主题，对标创建标准和要求，坚持以升促建，加快创建工作。加强基础设施建设，探索建立“一区多园”管理模式，推动高新区建设协同联动、多点开花。依托珠海清华科技园在高新区高标准建设孵化园区，鼓励发展众创、众包、众扶、众筹等新型孵化模式，引进入孵企业20家以上，力争创建成国家级孵化器。加强市五金刀剪产业技术研究院建设。加快组建产学研创新联盟，建成阳江市五金刀剪产业协同创新中心。以推动产业升级为主攻方向，以“六大产业”龙头企业为主导，实施核心技术攻关项目。

　　构建技术创新体系。推动行业龙头企业创建国家级重大创新平台实现突破，新创建省级工程研究中心4家、市级工程研究中心20家，全市规上企业研发机构覆盖率达25%以上。积极培育高新技术企业，争取新入库企业30家、存量企业突破60家。开展质量提升行动，完善公共检测服务平台，建设广东省质量监督不锈钢复合材料检验站，新增省名牌产品12个，争取新立项国家或行业标准1项。

　　营造良好创新生态。推动产学研深度融合、科技创新与标准化生产深度融合，针对我市产业特色，积极对接国内高等院校和科研院所，提高科技创新能力。规划建设科技创新园。推进国家知识产权试点城市建设，加快建设中国阳江(五金刀剪)知识产权快速维权中心。利用市人才驿站等平台，加大柔性引才力度。积极引进各类创投机构，联合设立产业发展基金，建立多渠道科技金融服务体系。支持发展数字经济、共享经济，培育新动能。

　　(六)开创改革开放新局

　　深化“放管服”改革。推进行政审批制度改革，及时调整完善权责清单、审批事项，全面推行并联审批、容缺预审。加快“一门式一网式”政府服务模式改革，完善市级改革模式，年内完成县(市、区)、镇(街道)、村(社区)三级政务服务模式改革任务，实现“一门在基层、服务在网上”。加快数字政府建设，解决政务信息化建设“各自为政、条块分割、信息孤岛”问题。进一步清理减少行政职权，深入开展“减证便民”专项行动。加快政府职能转变，深化行政管理体制改革，优化部门机构和职能配置。推进事业单位改革，扩大事业单位自主管理权限。

　　深化经济领域改革。减少投资项目前置审批，推行PPP模式，充分调动社会资本参与重要基础设施、重点产业项目建设积极性。扎实推进市以下财政事权和支出责任划分改革试点，探索建立事权和支出责任相适应的制度，改变预算控制方式，全面加强绩效管理。深入推进“证照分离”改革和个体工商户登记制度改革试点，激发创新创业活力。健全社会信用体系，完善守信激励和失信惩戒机制。深化国有企业改革，推动城投等四大国企突出主业、增强实力。完成国有林场、盐业体制改革。

　　深化区域合作交流。抓住省在更高起点上持续推动粤东西北地区加快发展的机遇，主动对接珠海对口帮扶，在园区产业共建、城市扩容提质、民生事业发展、改革开放探索、精准扶贫攻坚上取得新成效。主动融入粤港澳大湾区，积极参与“珠中江阳”经济圈建设，联合推动跨界交通基础设施、珠江西岸先进装备制造产业带建设和环境共治等方面合作。促进湛茂阳沿海经济带加快发展，积极争取北部湾城市群发展规划政策红利。主动承接广东自贸区的辐射带动，积极参与“一带一路”建设。

　　(七)提升生态环境优势

　　加强生态保护建设。牢固树立绿水青山就是金山银山的理念，推进国家环保模范城市和国家森林城市创建工作，促进人与自然和谐共生，让绿色成为城乡人居环境的底色。深入实施新一轮绿化阳江大行动，全年造林更新面积6万亩以上。加强海洋生态保护，积极建设“蓝色海湾”和“美丽海湾”，严格保护岸线、海岛和湿地，新种红树林600亩以上。落实城市总体规划和土地利用总体规划，强化建设用地空间管制，推行能源和水资源消耗、建设用地等总量和强度“双控”，提高资源节约利用水平。加强矿山地质环境保护和恢复治理。严守耕地保护红线，完成垦造水田任务。

　　加强环境综合整治。以改善环境质量为核心，坚决打好污染防治攻坚战。全面实施城市空气质量达标管理，重点加强臭氧和PM2.5治理，坚决打赢蓝天保卫战。深入实施水污染防治行动计划，加强农业面源污染防治，推进禁养区、禁建区畜禽养殖场整治，加快清理整治水源保护区违法违规建筑，完成省下达的黑臭水体整治任务。努力构筑平安绿色生态水网，落实河长制、湖长制，推进漠阳江流域综合整治，年内动工建设漠阳江出海口综合治理工程和大河水库引水工程。统筹推进城乡生活污水治理，抓好中洲岛、三江岛污水处理厂和11个中心镇、24个一般镇污水处理设施及配套管网建设。推进土壤污染治理，加强固体废物管理。全面开展“小散乱污”企业专项整治，确保城镇生活垃圾无害化处理率达100%。

　　加强环保执法，落实生态环境损害赔偿制度改革方案。继续抓好中央环境保护督察整改，开展第二次全国污染源普查。

　　加强节能减排降耗。严格项目环保准入，推进重点企业强制性清洁生产审核，加快建立绿色低碳循环发展的经济体系。推广新能源汽车及装备，新投放100辆新能源公交车，推进电动汽车充电基础设施建设。制定落实节能整改清单，严格执行能耗限额标准，通过实施差别电价等措施，加强电力、钢铁和五金刀剪等重点行业和重点用能企业节能管理，倒逼企业实施能效提升工程。大力发展循环经济，推进园区循环化改造，促进企业清洁生产，实现资源综合利用。

　　(八)提高保障民生水平

　　强化就业社会保障。落实就业优先战略和积极就业政策，城镇新增就业3.5万人，就业困难人员实现再就业2000人。扎实推进机关事业单位养老保险制度改革，实现新老制度平稳过渡。抓好社会保险扩面征缴，提升社会保险待遇水平。加强城乡低保、特困供养等社会救助，落实残疾人权益保障，关爱农村留守儿童。推进县级养老设施建设，积极探索社区居家养老模式。促进房地产市场平稳健康发展，加快建立多主体供给、多渠道保障、租购并举的住房制度，新建保障性住房400套。

　　办好教育卫生事业。优先发展教育事业，年内创建成教育现代化先进市，努力让每个孩子都能享有公平而有质量的教育。实施第三期学前教育行动计划，增加公立幼儿园数量，扩大普惠性学前教育资源。新建10所义务教育学校，解决城镇普通中小学大班额问题。推动阳江技师学院、阳江第一职业技术学校等建成特色职业名校。统筹办好高等教育、特殊教育、继续教育，促进民办教育健康发展，扎实推进阳江职业技术学院新校区规划建设。深入实施“强师工程”，推进义务教育学校教师县管校聘改革。加强校园安全管理。推进卫生强市建设，深化医药卫生体制改革和计划生育服务管理改革，探索建立现代医院管理制度，大力推进医联体模式，在阳东区、阳春市各建一个医共体试点。建立健全分级诊疗制度，县域内就诊率提高到90%左右，基层医疗卫生机构诊疗量占总诊疗量比例达到65%以上。抓好市中医院二期、市保健院二期建设和市第三人民医院搬迁工程，升级建设县级公立医院和两家镇中心卫生院，推进社区卫生服务中心和村卫生站标准化建设。完善市县两级院前急救体系，丰富家庭医生健康管理、个性化服务等内涵，提高签约率。推动中医药振兴发展。加强医疗学科与队伍建设。深入开展爱国卫生运动，倡导文明生活方式，新创建省卫生村50条。

　　推动文化繁荣发展。践行社会主义核心价值观，深入创建全国文明城市，大力提高群众文明素养和全社会文明程度。以城市建成“十分钟文化圈”和农村建成“十里文化圈”为目标，进一步健全市、县、镇、村四级公共文化设施网络。推进市体育综合训练馆建设，完成漠阳楼、风筝馆改造工程。大力繁荣社会主义文艺，办好风筝文化节、南国书香节暨阳江书展、“蚝美阳江”美食节等活动，加强文化下乡惠民服务，丰富人民群众文化生活。开展全民健身活动，抓好帆船帆板、海陵岛环岛马拉松赛等特色体育品牌项目。加快海丝文化名城建设，推进海上丝绸之路等文化遗产开发，深入开展“南海I号”发掘保护工作，积极参与海上丝绸之路世界文化遗产申报。大力发展文化产业，规划建设文化创意产业园，扶持漆艺、风筝等传统工艺的传承发展。新建市档案馆(地方志、党史馆)，完成自然村落历史人文普查。

　　创新社会治理格局。加强社区治理体系建设，推动社会治理重心向基层下移。落实“七五”普法规划，做好司法行政工作。加强平安阳江建设，抓好综治信访维稳，加强社会矛盾排查化解，推进各级综治“中心+网格化+信息化”和“雪亮工程”建设，严密防范和严厉打击各类违法犯罪活动。强化反走私综合治理，提高管边控海能力。完善各类应急预案，加快大应急平台建设。强化安全生产责任体系建设，加大隐患排查治理力度，坚决遏制重特大事故发生。加强食品药品安全监管，建立健全食品药品质量追溯制度。做好国防动员、国防教育、人民防空、国家安全等工作，落实双拥优抚安置政策，推动军民融合深度发展，完善复退军人服务体系建设。发展民族宗教、外事侨务、对台事务等事业，支持工会、共青团、妇联、残联、科协等开展工作。

　　加快补齐民生短板。对标决胜全面建成小康社会，巩固提升已达标的16项指标，扎实推进达到时序进度的12项指标，加快提升11项短板指标。投入39亿元，集中力量办好涵盖底线民生保障、扶贫济困、文化教育、医疗卫生、农村生产生活条件、基层基本公共服务、环境整治、就业创业、宜居城乡建设等方面的十件民生实事。民生实事是一面镜子，折射的是政府部门的为民情怀;民生实事是一道考题，考验的是全市干部的责任担当，我们将带着情怀和责任，一件件解决好，一桩桩落实好，以实实在在的成效兑现我们的承诺，使改革发展成果惠及全体人民，不断增强人民群众的获得感、幸福感。

　　三、加强政府自身建设

　　(一)增强政治意识，维护党的领导。认真开展“不忘初心、牢记使命”主题教育，以习近平新时代中国特色社会主义思想武装头脑、指导实践、推动工作。旗帜鲜明讲政治，增强“四个意识”，坚定“四个自信”，自觉维护党中央权威和集中统一领导，在思想上政治上行动上同以习近平同志为核心的党中央保持高度一致。严守政治纪律和政治规矩，坚决贯彻中央、省委和市委的各项决策部署，在市委的领导下开展工作。

　　(二)树立法治思维，推进依法行政。切实将政府活动全面纳入法治轨道，坚持法定职责必须为、法无授权不可为。自觉接受人大监督和政协民主监督，认真办理人大代表议案建议和政协提案。完善重大事项决策程序，加强规范性文件合法性审查，落实政府法律顾问制度，提高决策科学化、民主化、法治化水平。全面推进政务公开，坚持严格规范公正文明执法，深化行政复议体制改革。

　　(三)加强业务学习，提高履职本领。针对“本领恐慌”“能力不足”的问题，大兴学习之风，建设学习型政府。各级领导干部要结合岗位职责，有针对性地学习业务知识，坚持理论联系实际，真正做到谋划推动工作“既要有想法、更要有办法”，不断提高领导能力和决策水平。建设高素质专业化干部队伍，提升各级干部的专业思维、专业素养和专业能力，适应新时代要求。

　　(四)深入转变作风，敢于担当作为。作风建设永远在路上。认真贯彻中央八项规定和实施细则，驰而不息纠“四风”。坚决破除思维定势、工作惯性和路径依赖，对定下来的事立说立行、马上就办、办就办好，雷厉风行抓落实。大兴调查研究之风，深入开展“大学习、深调研、真落实”活动。建立健全正向激励和容错纠错机制，加大对懒政怠政行为的追责问责力度，严厉整肃行政不作为、慢作为、乱作为，让敢于担当、善于担当蔚然成风。

　　(五)强化正风肃纪，建设廉洁政府。认真落实全面从严治党主体责任和“一岗双责”，深入推进政府系统党风廉政建设，营造风清气正的良好政治生态。强化监察、审计监督，加大公共资金、国有资产、国有资源监管力度。保持惩治腐败高压态势，聚焦重点领域，严肃查处侵害群众利益的不正之风和腐败问题，进一步树立干净干事、清正廉洁的政府形象。

　　各位代表!新时代要有新气象，新使命要有新作为。让我们紧密团结在以习近平同志为核心的党中央周围，在省委、省政府和市委的正确领导下，不忘初心、牢记使命，团结一心、凝聚力量，朝着“决胜全面小康、建设富美阳江”的目标阔步前进，为广东建设成为向世界展示习近平新时代中国特色社会主义思想的重要“窗口”和“示范区”作出应有的贡献，以优异成绩向改革开放40周年献礼!
